# Assisted reproductive technology: prevalence and associated factors in Southern Brazil

**DOI:** 10.11606/S1518-8787.2019053000737

**Published:** 2019-01-18

**Authors:** Shana Ginar da Silva, Andréa Dâmaso Bertoldi, Mariângela Freitas da Silveira, Marlos Rodrigues Domingues, Kelly R Evenson, Iná Silva dos Santos

**Affiliations:** I Universidade Federal de Pelotas. Faculdade de Medicina. Programa de Pós-Graduação em Epidemiologia. Pelotas, RS, Brasil; II Universidade Federal de Pelotas. Faculdade de Medicina. Departamento de Medicina Social. Programa de Pós-Graduação em Epidemiologia. Pelotas, RS, Brasil; III Universidade Federal de Pelotas. Escola Superior de Educação Física. Departamento de Desportos. Programa de Pós-Graduação em Educação Física. Pelotas, RS, Brasil; IV University of North Carolina at Chapel Hill. Gillings School of Global Public Health. Department of Epidemiology. NC, United States

**Keywords:** Reproductive Techniques, Assisted, Fertilization in Vitro, Embryo Transfer, Insemination, Artificial, Risk Factors, Socioeconomic Factors, Low-Middle Income Countries

## Abstract

**OBJECTIVE:**

To assess the prevalence of successful assisted reproductive technology and to identify the associated factors.

**METHODS:**

This population-based birth cohort study was carried out with 4,333 pregnant women expected to deliver in 2015 in the urban area of Pelotas, Southern Brazil. Use of an assisted reproductive technology procedure, type of assisted reproductive technology [in vitro fertilization or intracytoplasmic sperm injection or artificial insemination], number of embryos transferred, success of embryo transfer, number of attempts, and reported reasons for seeking assisted reproductive technology were the main outcomes measured. Use of an assisted reproductive technology procedure was analyzed according to sociodemographic, nutritional, reproductive history, and behavioral characteristics. Unadjusted and adjusted analyses were performed by logistic regression.

**RESULTS:**

Among the 4,275 newborns enrolled in the Pelotas 2015 Birth Cohort Study, 18 births (0.4%) were conceived by assisted reproductive technology. Most cases of assisted reproductive technology were by in vitro fertilization (70.6%). All cycles were performed in private clinics under direct out-of-pocket payment. Even after controlling for confounders, maternal age > 35 years, nulliparity and high family monthly income were strongly associated with assisted reproductive technology.

**CONCLUSIONS:**

The use of assisted reproductive technology services was reported by only a few women in the Pelotas 2015 Birth Cohort Study. Our study highlights sociodemographic factors associated to assisted reproductive technology procedures. To better understand the patterns and barriers in overall use of assisted reproductive technology services over time, national-level trend studies in assisted reproductive technology treatments and outcomes, as well as studies exploring the characteristics of women who have sought this kind of treatment are needed in low-middle income countries.

## INTRODUCTION

Infertility is one of the main reproductive health disorders affecting a high proportion of the population worldwide^1^. Global infertility prevalence rates are difficult to estimate, due to multiple factors. However, according to the Demographic and Health Survey, one in every four couples in low-middle income countries are affected by infertility^1^. It has been estimated that the number of infertile people in the world may be as high as 15%, particularly in industrialized nations^2^. A systematic analysis of national, regional, and global trends in infertility in more than 190 countries and regions around the world estimated that, in 2010, 48.5 million couples worldwide were infertile^3^. In addition, it is estimated that the prevalence of infertility will grow in the coming years, considering that lifestyle factors such as alcohol consumption, smoking, obesity, lack of physical activity, and sexually transmitted diseases, which interfere negatively in female and male fertility, are increasing in the general population^4^.

Since the first baby with assisted reproductive technology (ART) was conceived in 1978, the use of advanced technologies to overcome infertility has increased steadily^5^. Children conceived by ART comprise as many as 5.9% of total births in Denmark^6^, 4.2% in Israel^7^, 3.3% in Australia^8^, 1.6% in the United States^9^, 1.5% in Japan^10^, and 1.7%–2.2% in the largest European countries^11^. In 2014 in the United States, 169,568 ART procedures resulted in 56,028 live-birth deliveries and 68,782 infants, representing 1.6% of births for that year^5^.

Infertile patients are having the opportunity to realize their dreams of obtaining a family through ART. However, the availability of ART services varies around the world. European countries accomplish approximately 55% of all the ART cycles in the world, North America 20%, Asia 10%, Middle East 6%, Australia/New Zealand 6%, and Latin America 3%^12^. According to the Latin American Network of Assisted Reproduction, most initiated cycles of ART were reported by Brazil, representing 44% of all cycles, followed by Argentina and Mexico, with 23% and 13% respectively^13^. In 2013, data from the 7th Report of the Brazilian Embryo Production System showed that 24,147 initiated cycles of ART were produced with important differences between Brazilian regions: 66% of these procedures were performed in ART clinics situated in the Southeast region and only 1% in the Northern region of the country^14^. However, with access to 512 cycles per million women aged 15–45 years, Brazil is far behind high-income countries^13^.

The nature of the healthcare system, economics, relative cost of treatment, availability of high-technology services, and government regulations are aspects that may influence access to ART^12^. In 2012, the Brazilian government launched a policy establishing ART as a universal right within the National Health System^15^. An increase in ART coverage is expected after substantial economic support. However, comprehensive epidemiological population-based studies on the prevalence and correlates are scarce and not well documented in low-middle income countries such as Brazil.

To investigate the use as well as characteristics of the population in which these ART procedures are performed are of vital importance for the planning and monitoring of reproductive health and maternal-child health policies. Thus, the purpose of this study was to assess the prevalence of ART and to identify the associated factors in a population-based birth cohort study in Southern Brazil.

## METHODS

### Setting and Study Design

A population-based birth cohort study was conducted with pregnant women expected to deliver in 2015 in the urban area of Pelotas. Pelotas is a city in Southern Brazil with approximately 320,000 inhabitants situated 170 miles from Porto Alegre, the state capital. The Human Development Index of Pelotas is considered high (0.74), the Gini Index is 0.54 and the illiteracy rate is less than 5%^16^. In 2015, the infant mortality rate in Pelotas was 13.3 deaths/1,000 live births, similar to the overall country^17^.

Pregnant women were recruited from all health facilities offering antenatal care (public and private). Women contacted before 16 weeks of pregnancy were interviewed at enrollment (initial assessment) and between the 16^th^ and the 24^th^ week of pregnancy (main assessment). Women not identified before the 16^th^ week responded to a ‘combined assessment’ tool that consisted of a combination of the information collected in the ‘initial’ and ‘main assessments’.

Thereafter, mothers were interviewed at the hospital soon after delivery (perinatal study). All five maternity hospitals from both private and public insurance were visited daily from January 1 to December 31 2015, and all births from mothers living in the urban areas of the city were identified. Face-to-face interviews took place in the hospital within 48h after the delivery. Most mothers interviewed at the hospital (73.8%) had already been enrolled during pregnancy. Further methodological details of the 2015 Pelotas Birth Cohort Study can be found elsewhere^18^.

The study was approved by the Ethics Committee of the Physical Education School at the Universidade Federal de Pelotas in an official letter numbered 522/064. Written informed consent was obtained from all participants.

### Outcome and Related Characteristics

Information on ART procedures were gathered at the antenatal and the perinatal interviews. Utilization of ART procedures was evaluated using the question: “Did you have an artificial fertilization in this pregnancy?” Women who answered ‘yes’ were contacted later (during 2017) and were invited to take part in a sub-study.

Women in the sub-study were interviewed by phone. After five failed attempts of a phone interview, women were interviewed at home. The use of an ART procedure was firstly confirmed and then other related characteristics, such as type of ART [in vitro fertilization (IVF) or intracytoplasmic sperm injection (ICSI)], number of embryos transferred, success of embryo transfer, number of attempts, and reported reasons for seeking ART.

Conceptually, ART does not include assisted insemination (artificial insemination) using sperm from either the woman's partner or a sperm donor^12^. However, due to the almost absolute absence of official information on ART in Brazil, we decided also to include artificial insemination as part of our outcome.

Information on sociodemographic, nutritional, reproductive history, and behavioral variables was gathered at the interview carried out in the hospital. The correlates were defined as follows: age (< 30; 30–35; 36–39; ≥ 40 years), skin color (white or brown/black), marital status (living with or without a partner), parity (1; ≥ 2), family income (1–3; 3.1–10; > 10 minimum wages) (1 minimum wage was equivalent to US$300.00), maternal schooling (0–8; 9–11; ≥ 12 completed years), paid job during pregnancy (yes, no), smoking during pregnancy (yes, no), alcohol use during pregnancy (yes, no), history of miscarriage (yes, no), history of preterm birth (yes, no), irregular menstrual cycles three months before pregnancy (yes, no), and leisure-time physical activity (LTPA) three months prior to pregnancy (< 150; ≥ 150 minutes/day) measured by a questionnaire developed by researchers from the Pelotas 2004 Birth Cohort Study^19^. Pre-pregnancy body mass index (BMI) was calculated by dividing weight by height squared (kg/m^2^)^20^.

Two types of data quality control were carried out during the perinatal study. The first one was an informal daily-based hospital visit provided by fieldwork supervisors to a randomly chosen sample of mothers. In addition, a data quality control by phone contact was performed in 10% of the total interviews using a short questionnaire.

### Statistical Analysis

**Figure 1:**
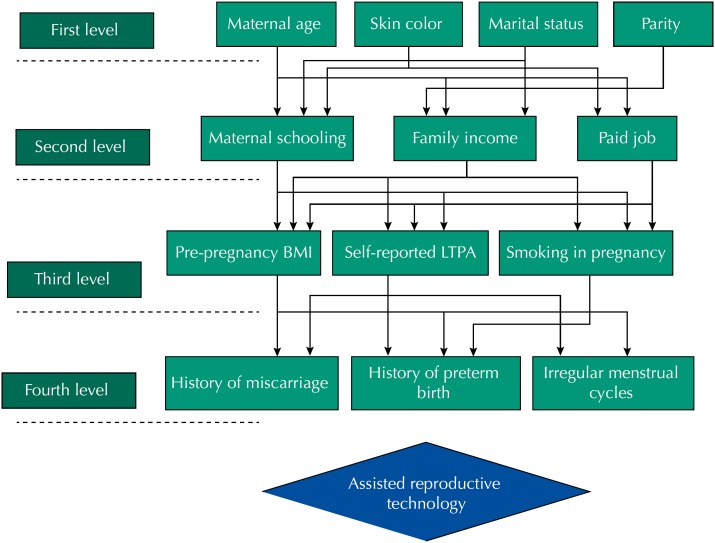
Hierarchical levels of analysis based on a conceptual model.

Descriptive analyses are presented in relative (%) and absolute frequencies (n). Chi-square test and Fisher's exact test were used to compare differences between groups. Unadjusted and adjusted analyses were performed by logistic regression. Adjusted analysis was performed using hierarchical levels^21^ based on a conceptual framework built by the authors (Figure 1). For the selection of variables, the backward method was applied within each level and variables with p < 0.20 were kept in the model. The hierarchical model consisted of four levels. The first level was composed by demographic variables: maternal age, skin color, marital status and parity. The second level comprised socioeconomic variables (maternal schooling, family income and paid job during pregnancy). At the third level, nutritional and behavioral variables were included: pre-pregnancy BMI, self-reported LTPA and smoking during pregnancy. The fourth level was composed of history of miscarriage, preterm birth and irregular menstrual cycles three months before pregnancy. The variables included in the adjusted model may be related to problems such as hormonal dysfunctions, low quality of ovulation, obstructed tubes and endometriosis that can lead to infertility, and then can lead the women to perform the ART.

Statistical significance was set at 5%, and 95% confidence intervals were adopted. All analyses were performed using the software Stata version 12.1 (StataCorp, College Station, Texas, USA).

## RESULTS

A total of 4,333 live births took place in Pelotas in 2015 from mothers living in the urban area of the city. Losses and refusals to participate in the perinatal study accounted for 1.3% of the deliveries. Among the 4,219 births enrolled in the 2015 Birth Cohort Study, 18 (0.4%) were conceived by ART.

Only one of the 18 ART-mothers refused to participate in the sub-study. Most ART cases were by IVF (70.6%; Table 1). Most women had transferred two embryos (91.7%) and had success of embryo transfers in the first attempt (58.8%). The most frequent reason for seeking ART was low quality of sperm (23.5%) followed by obstructed tubes (17.7%), unexplained infertility (17.7%), and other reasons (17.7%). More than half of the women (52.9%) were submitted to ovulation induction therapy before ART.

**Table 1:** Characteristics of the assisted reproductive technology procedures. 2015 Pelotas (Brazil) Birth Cohort Study.

Variable		n	%
Type of ART			
	In vitro fertilization (IVF)	12	70.6
	Artificial insemination (AI)	3	17.7
	Intracytoplasmic sperm injection (ICSI)	2	11.7
Number of embryo transfers (IVF/ICSI)			
	1	1	8.3
	2	11	91.7
Success of embryo transfers in the first time			
	Yes	10	58.8
	No	7	41.2
Number of attempts in ART			
	2	3	42.9
	3	1	14.3
	4	2	28.6
	5	1	14.2
Reported main reason for seeking ART			
	Endometriosis	2	11.8
	Polycystic ovary syndrome	1	5.9
	Low quality of ovulation	1	5.9
	Obstructed tubes	3	17.7
	Unexplained infertility	3	17.7
	Low quality of sperm	4	23.5
	Other	3	17.7
Treatment with ovulation induction before ART			
	Yes	9	52.9

ART: assisted reproductive technology

Note: 1 mother refused to provide information.

Most of the IVF/ICSI and artificial insemination procedures were made in clinics situated in Porto Alegre (the state capital), while two cycles were made in clinics in São Paulo and two in clinics located in Pelotas. The ART procedures were not paid by the Brazilian Unified Health System. All cycles were performed in private clinics under direct out-of-pocket payment.

In Table 2, characteristics of ART-mothers are compared to characteristics of mothers with spontaneous pregnancy in the cohort. ART-mothers were in general older (p < 0.001), had a higher level of education (p < 0.001) and higher family income (p < 0.001) than mothers who had spontaneous pregnancies. Nulliparity (p < 0.001), paid job during pregnancy (p = 0.02), and pre-pregnancy engagement in LTPA (p = 0.008) were more prevalent among ART-mothers. There were no black-skin mothers at the ART group.

**Table 2:** Maternal characteristics among ART and spontaneous pregnant women in the 2015 Pelotas (Brazil) Birth Cohort Study.

Variable		Spontaneous pregnancy		ART		p
		(n = 4,201)		(n = 18)		
		n	%	n	%	
Age (years)						< 0.001
	< 30	2,611	62.2	2	11.1	
	30–35	1,122	26.7	8	44.4	
	36–39	345	8.2	5	27.8	
	> 40	122	2.9	3	16.7	
Skin color						0.30
	White	2,967	70.8	15	83.3	
	Other	1,227	29.3	3	16.7	
Marital status						0.50
	Living with a partner	3,602	85.8	17	94.4	
	Living without a partner	598	14.2	1	5.6	
Parity						0.001
	1 (primaparae)	2,068	49.3	16	88.9	
	≥ 2	2,131	50.7	2	11.1	
Schooling (years)						< 0.001
	0–8	1,470	35.0	1	5.6	
	9–11	1,440	34.3	1	5.6	
	12+	1,289	30.7	16	88.9	
Family income						< 0.001
	1–3	2,521	60.0	4	22.2	
	3.1–10	1,425	33.9	5	27.8	
	> 10	253	6.0	9	50.0	
Paid job during pregnancy						0.02
	Yes	2,328	55.4	15	83.3	
Pre-pregnancy BMI (kg/m^2^)						0.86
	Normal	2,119	52.5	10	55.6	
	Overweight	1,132	28.1	4	22.2	
	Obese	783	19.4	4	22.2	
History of miscarriage						0.66
	Yes	347	8.3	2	11.1	
History of preterm birth						0.40
	Yes	991	23.6	6	33.3	
Menstrual irregular cycle three months before pregnancy						0.61
	Yes	1,170	28.0	6	33.3	
Smoking during pregnancy						0.34
	Yes	697	16.6	1	5.6	
Self-reported pre-pregnancy LTPA (minutes/week)						0.008
	≥ 150	667	15.9	7	38.9	

ART: assisted reproductive technology; BMI: body mass index; LTPA: leisure-time physical activity

Correlates of ART were further investigated in a multivariable model to control for potential confounding (Table 3). Even after controlling for confounders, age, parity and income were very strong correlates of ART. Women older than 35 years were six times more likely to have used ART as women aged 35 years or younger. Nulliparous women were 10 times more likely to have used ART than parous women. Wealthier women were 3.7 times more likely to have used ART services compared to women from the lowest income group (1–3 minimum wages). The crude association with schooling and LTPA was not confirmed in adjusted analysis.

**Table 3:** Unadjusted and adjusted association between maternal characteristics and ART in the 2015 Pelotas (Brazil) Birth Cohort Study.

Variable		Crude			Adjusted*		
		OR	95%CI	p	OR	95%CI	p
Level 1							
Age (years)				< 0.001			< 0.001
	≤ 35	Ref	Ref		Ref	Ref	
	> 35	6.4	2.5–16.3		15.7	5.9–41.6	
Skin color				0.25			
	White	2.1	0.6–7.2		–	–	
	Other	Ref	Ref				
Marital status				0.31			
	Living with a partner	2.8	0.4–21.2		–	–	
	Living without a partner	Ref	Ref				
Parity				0.005			< 0.001
	1 (primaparae)	8.2	1.9–35.9		18.6	4.1–85.2	
	≥ 2	Ref	Ref		Ref	Ref	
Level 2							
Schooling (years)				< 0.001			0.05
	< 12	Ref	Ref		Ref	Ref	
	≥ 12	18.1	4.1–78.7		5.1	1.0–25.6	
Family monthly income				0.003			0.02
	1–3	Ref	Ref		Ref	Ref	
	3.1–10	2.2	0.6–8.2		0.7	0.2–2.9	
	> 10	22.4	6.9–73.3		3.7	0.9–14.8	
Paid job during pregnancy				0.03			
	Yes	4.0	1.2–13.9		–	–	
	No	Ref	Ref				
Level 3							
Pre-pregnancy BMI (kg/m^2^)				0.80			
	Normal	Ref	Ref				
	Overweight/Obese	0.9	0.4–2.2		–	–	
Smoking during pregnancy				0.24			
	Yes	0.3	0.04–2.2		–	–	
	No	Ref	Ref				
Self-reported pre-pregnancy LTPA (minutes/week)				0.01			
	≥ 150	Ref	Ref				
	< 150	3.4	1.3–8.7		–	–	
Level 4							
History of miscarriage				0.66			
	Yes	1.4	0.3–6.1		–	–	
	No	Ref	Ref				
History of preterm birth				0.36			0.15
	Yes	1.6	0.6–4.2		2.1	0.8–6.0	
	No	Ref	Ref		Ref	Ref	
Menstrual irregular cycle three months before pregnancy				0.62			
	Yes	1.3	0.5–3.4		–	–	
	No	Ref	Ref				

ART: assisted reproductive technology; BMI: body mass index; LTPA: leisure-time physical activity; Ref: reference category

* Adjusted for age, parity, schooling, income and history of preterm birth.

## DISCUSSION

This study documents the prevalence and the correlates of successful ART users in a population-based birth cohort study in Southern Brazil. Less than 1% of the total newborns in the Pelotas 2015 Birth Cohort were conceived by ART. IVF was the most used type of ART, with most women transferring two embryos. Age, parity, education and family income were strongly associated with ART.

Our study showed that 0.4% of the total live births in the 2015 cohort study resulted from ART techniques. This proportion is lower in comparison with others from high-income countries reported in the literature^6^ ^,^ ^7^, but close to the prevalence observed in the United States and Japan^10^ ^,^ ^11^. Data from the Latin American Network of Assisted Reproduction showed that Brazil is the country with the highest ART cycles in Latin America with 56 clinics providing ART services around the country in 2013. Nevertheless, the access is far behind high-income countries^13^. Also, there are high disparities between Brazilian regions because most procedures take place in the Southeast and South regions. Reasons for the disparities between countries and regions in use of ART procedures may include access barriers such as the high cost of medical services for infertility and the lack of adequate health insurance to afford the necessary diagnostic or treatment services^22^. Another reason is that governing authorities in low- and middle-income countries face different public health problems, leading them to place lower priority on ART availability.

**Figure 2:**
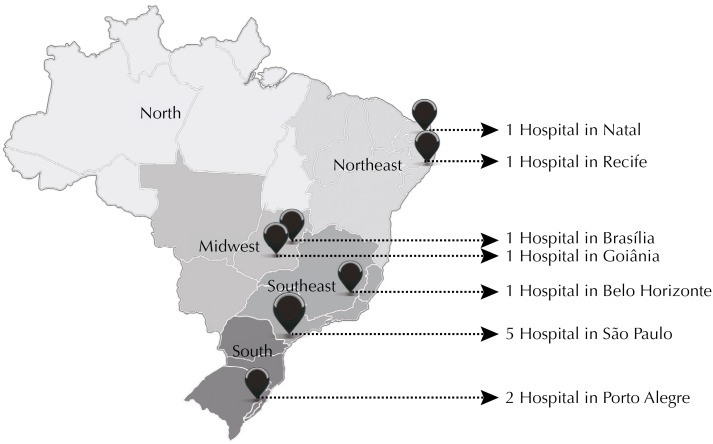
Spatial distribution of the 12 clinics that provide assisted reproductive technology (ART) by the Brazilian Unified Health System.

Several factors can affect the access to ART, such as culture, religion, political characteristics, and cost of treatment. The nature of the healthcare system, the availability of high technology services and the insurance coverage, the government regulations and the professional guidelines are other factors that can influence access to ART services. Besides all the regional-specific and social differences, the availability of ART critically varies according to the public vs. private funding model that is in place^23^. In 2012, the right to start a family was embraced by the Brazilian Unified Health System^15^ as a human right. Since then, 12 clinics and hospitals received financial support from the Brazilian government to provide universal access to ART services (Figure 2). As shown in the map, most of these clinics are situated in the city of São Paulo, without clinics in the North region of Brazil. As the financial support and the availability of free services increase, the inequalities in ART procedures are expected to decrease^15^. However, in our study the women reported that all expenses with ART procedures were covered by themselves. We found no official surveillance data to assess whether there were any changes in the coverage of ART since the implementation of the 2012 Brazilian new policy.

Similarly to previous studies^23^ ^,^ ^24^, our findings showed that age, parity and family income are strongly associated with ART procedures. Most of ART cycles were performed in nulliparous women aged 30–35 years. Previous studies have shown that women who make treatment for infertility tend to be a highly selected group, which may reflect the fact that women from lower socioeconomic status are less likely to have adequate health insurance coverage and other financial resources to afford the necessary diagnostic or treatment services^22^ ^,^ ^24^ ^-^ ^26^.

Our study showed that more than 90% of women transferred two embryos during ART procedure. This finding is similar to previous studies^24^. Because treatments are expensive and often are not covered by insurance plans, one approach to increase the potential success of any cycle leading to a live birth is to transfer multiple embryos during an IVF procedure^24^. However, due to the increased risk of adverse birth outcomes in multiple pregnancies, a healthy singleton birth is the ideal outcome for an ART procedure. Since 1996, the practice of embryo transfer decreased from 4+ embryos to 2 embryos in 2010^27^ ^,^ ^28^.

To the best of our knowledge, this is the first study to assess the prevalence of births resulting from ART and its correlates in a large and representative population sample in Brazil. However, some limitations should be noted. Our study captured only women who were successful in the procedure with live births resulting from ART. However, even in the United States, where the number and proportion of women undergoing ART have increased consistently over time, the number of births resulting from ART procedures, as based on birth certificates, remains low at 1% or less of all births^21^. Thus, to better understand the overall scenery of ART, it would be important also to evaluate women who have used ART techniques, including those unsuccessful procedures.

## CONCLUSION

In summary, our study highlights the prevalence and the characteristics of successful ART procedures in a population-based study in Southern Brazil. Our findings may instruct health policy and planning as it demonstrates important sociodemographic disparities in women performing ART procedures. Whereas there is a policy establishing ART as a universal right within the National Health System, it is essential to implement surveillance data and monitoring system to assess the demand, coverage and characteristics of the women which reach out the fertility treatment within the Brazilian Unified Health System. Comprehensive coverage of ART can help increase access to fertility treatments. Additional research is needed to investigate the effects of ART on maternal-child health outcomes and unsuccessful procedures.
